# A genome-wide deletion mutant screen identifies pathways affected by nickel sulfate in *Saccharomyces cerevisiae*

**DOI:** 10.1186/1471-2164-10-524

**Published:** 2009-11-15

**Authors:** Adriana Arita, Xue Zhou, Thomas P Ellen, Xin Liu, Jingxiang Bai, John P Rooney, Adrienne Kurtz, Catherine B Klein, Wei Dai, Thomas J Begley, Max Costa

**Affiliations:** 1 New York University School of Medicine, Nelson Institute of Environmental Medicine, 57 Old Forge Road, NY 10987, USA; 2 Department of Biomedical Sciences, Gen*NY*Sis Center for Excellence in Cancer Genomics, University at Albany-State University of New York, 1 Discovery Drive, Rensselaer, New York, 12144 USA

## Abstract

**Background:**

The understanding of the biological function, regulation, and cellular interactions of the yeast genome and proteome, along with the high conservation in gene function found between yeast genes and their human homologues, has allowed for *Saccharomyces cerevisiae *to be used as a model organism to deduce biological processes in human cells. Here, we have completed a systematic screen of the entire set of 4,733 haploid *S. cerevisiae *gene deletion strains (the entire set of nonessential genes for this organism) to identify gene products that modulate cellular toxicity to nickel sulfate (NiSO_4_).

**Results:**

We have identified 149 genes whose gene deletion causes sensitivity to NiSO_4 _and 119 genes whose gene deletion confers resistance. Pathways analysis with proteins whose absence renders cells sensitive and resistant to nickel identified a wide range of cellular processes engaged in the toxicity of *S. cerevisiae *to NiSO_4_. Functional categories overrepresented with proteins whose absence renders cells sensitive to NiSO_4 _include homeostasis of protons, cation transport, transport ATPases, endocytosis, siderophore-iron transport, homeostasis of metal ions, and the diphthamide biosynthesis pathway. Functional categories overrepresented with proteins whose absence renders cells resistant to nickel include functioning and transport of the vacuole and lysosome, protein targeting, sorting, and translocation, intra-Golgi transport, regulation of C-compound and carbohydrate metabolism, transcriptional repression, and chromosome segregation/division. Interactome analysis mapped seven nickel toxicity modulating and ten nickel-resistance networks. Additionally, we studied the degree of sensitivity or resistance of the 111 nickel-sensitive and 72 -resistant strains whose gene deletion product has a similar protein in human cells.

**Conclusion:**

We have undertaken a whole genome approach in order to further understand the mechanism(s) regulating the cell's toxicity to nickel compounds. We have used computational methods to integrate the data and generate global models of the yeast's cellular response to NiSO_4_. The results of our study shed light on molecular pathways associated with the cellular response of eukaryotic cells to nickel compounds and provide potential implications for further understanding the toxic effects of nickel compounds to human cells.

## Background

The sequencing of the human and yeast genomes and the high conservation in gene function found between yeast genes and their human homologues has made *Saccharomyces cerevisiae *a fast and cost-efficient model organism to deduce biological processes in human cells. Data from genomic analysis of yeast transcriptional profiling, yeast two-hybrid screen, cellular localization, and transcription factor binding studies have provided a very thorough understanding of the biological function and regulation of the yeast genome and proteome, as well as allowed computational methods to generate global models of the cellular responses to environmental agents [1]. Deletion mutations of *S. cerevisiae *constructed for ~6200 known genes have identified ~4733 viable haploid gene-deletion mutants [2-4]. Genome-wide phenotyping screens, that screen deletion mutants of the entire set of nonessential yeast genes, have been useful in the past to elucidate the role of nonessential proteins in modulating toxicity after exposure to DNA damaging agents and environmental stressors [1,5-10]. Additionally, data from genomic phenotypic screens have allowed for the generation of cellular recovery pathways by merging global phenotypic data with global cellular localization and protein interactome data [1]. This type of analysis has been a useful method to shed light on previously little known molecular mechanisms/pathways associated with the tolerance of eukaryotic cells to toxic agents.

Nickel (Ni) is a toxic and carcinogenic metal widely used in the production of coins, jewelry, stainless steel, batteries, certain medical devices, carbon nanoparticles, and in Ni refinery, plating and welding [11]. Occupational exposure to nickel compounds has been associated with respiratory distress and lung and nasal cancers [12]. Although epidemiological, animal, and cell culture studies have found nickel compounds to be carcinogenic [12-16], the precise mechanism(s) of nickel carcinogenesis remains unclear. Instead, numerous studies have implicated structural alterations in chromatin and epigenetic changes as the primary events in nickel carcinogenesis [17-26]. Nickel compounds have also been shown to interfere with cellular iron uptake and the function of enzymes containing iron in their active sites [27,28]. Other suggested mechanisms of nickel carcinogenesis include the inappropriate activation of several cellular stress response pathways involving MAPKs, PI3K, HIF-1, NFAT, and NF-κB (reviewed in [29].

We have completed a genome-wide phenotypic screen with a library of the entire set of nonessential haploid *Saccharomyces cerevisiae *gene deletion strains to assess the role of nonessential proteins in modulating toxicity upon exposure to NiSO_4_. Using our yeast genetic screen we have identified 149 genes whose gene deletion causes sensitivity to NiSO_4 _and 119 genes whose gene deletion confers resistance. Pathways analysis with proteins whose absence renders cells more sensitive and resistant to nickel identified a wide range of cellular processes engaged in the toxicity of *S. cerevisiae *to NiSO_4_. Functional categories overrepresented with proteins whose absence renders cells sensitive to NiSO_4 _include homeostasis of protons, cation transport, transport ATPases, endocytosis, siderophore-iron transport, homeostasis of metal ions, and the diphthamide biosynthesis pathway. Functional categories overrepresented with proteins whose absence renders cells resistant to nickel include functioning and transport of the vacuole and lysosome, protein targeting, sorting, and translocation, intra-golgi transport, regulation of C-compound and carbohydrate metabolism, transcriptional repression, and chromosome segregation/division. Seven nickel toxicity modulating networks and ten nickel resistance networks were identified. The biological function of the nickel toxicity modulating networks and resistance networks also highlight the pathways described above as well as identify components of the alkaline phosphatase pathway and THO nuclear complex as mediating sensitivity to nickel. Additionally, we studied the degree of sensitivity or resistance of the 111 nickel-sensitive and 72 resistant strains whose gene deletion product has a similar protein in human cells. In this study we suggest a possible role of the evolutionarily conserved diphthamide biosynthesis pathway as well as components of the outer kinetochore involved in chromosome segregation in mediating toxicity of *S. cerevisiae *to nickel.

We have carried out a genomic phenotypic screen in order to identify proteins in *S. cerevisiae *important for regulating cellular toxicity to nickel compounds and have used computational methods to integrate the data and generate global models of the yeast's cellular response to NiSO_4_. The results of our study shed light on molecular pathways important in the cellular response of *S. cerevisiae *to nickel compounds and provide potential implications for further understanding the toxic effects of nickel compounds to human cells.

## Results and Discussion

### Identification of nickel-sensitive and resistant *S. cerevisiae *single gene deletion mutants

**Figure 1:**
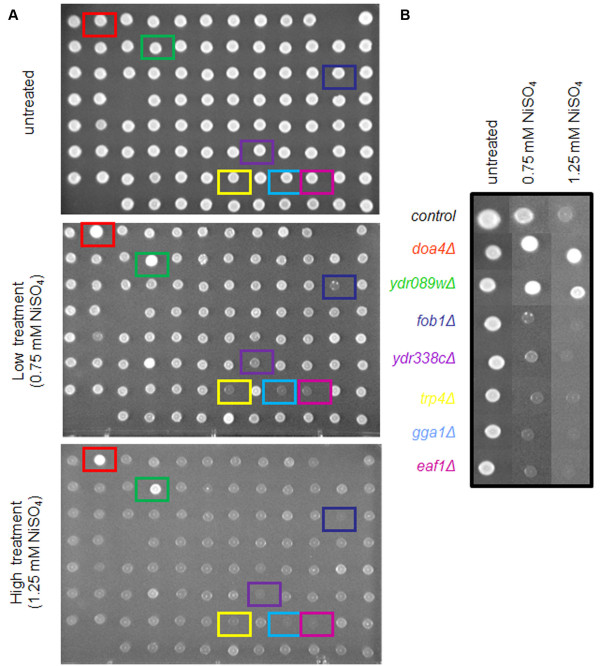
***S. cerevisiae *genomic phenotypic screen with NiSO_4_**. (A) Representative plate of the YPD-agar plates containing no NiSO_4 _(untreated), low (0.75), or high (1.25) concentration of NiSO_4 _spotted with 96 *S. cerevisiae *gene deletion mutant strains used in the genomic phenotypic screen. Dark blue, purple, yellow, light blue and pink squares identify the NiSO_4_-sensitive deletion strains *fob1Δ, ydr338cΔ, trp4Δ, gga1Δ, and eaf1Δ*, respectively. Red and green squares identify the NiSO_4_-resistant deletion strains *doa4Δ *and *ydr089wΔ*, respectively. (B) Recompiled images of nickel-sensitive and resistant identified on the plate in A. *doa4Δ and ydr089wΔ *are examples of how Ni-resistant mutant strains were identified, whereas the colony of the control strain changed color from white to gray with increasing concentrations of NiSO_4_, the resistant strains did not. The Ni-sensitive strains *fob1Δ, ydr338cΔ, trp4Δ, gga1Δ*, and *eaf1Δ *exhibited more of a growth defect compared to the control strain even under low (0.75 mM) NiSO_4 _concentration.

Nickel-sensitive and resistant strains were identified by screening the BY4741 *S. cerevisiae *strain and all of its single-gene deletion mutant derivatives corresponding to the complete set of 4733 nonessential yeast genes with a low (0.75 mM) and high concentration (1.25 mM) of NiSO_4_. Three replicates of the whole screen were done with fresh liquid master culture plates in 96 well plates. Strains were grown for 60 hrs at 30°C and then digitally imaged for analysis. Images of each plate were compiled and sensitive and resistant mutant strains were visually identified (Figure 1). Strains were labeled sensitive to NiSO_4 _if nickel treatment inhibited its growth. Strains were labeled resistant if nickel treatment did not inhibit its growth. This analysis identified 149 genes whose gene deletion causes sensitivity to NiSO_4 _and 119 genes whose gene deletion confers resistance. A complete list of the yeast systematic number of the proteins corresponding to the nickel-sensitive and -resistant gene deletion strains identified in our phenotypic screen is included in Additional file 1.

### Functional categories overrepresented with proteins whose absence renders cells sensitive to NiSO_4_

In order to obtain a more complete understanding of the cell's toxicity to nickel compounds and its mechanism(s) for regulating toxicity we assessed the cells' global response upon exposure to nickel. Functional categories overrepresented in our list of proteins whose absence renders cells sensitive or resistant to nickel were identified using FunSpec. Table 1 lists the seven functional categories overrepresented with proteins whose absence renders cells sensitive to NiSO_4_. MIPS functional categories overrepresented with mutants sensitive to nickel include homeostasis of protons, cation transport, siderophore-iron transport, homeostasis of metal ions (Na, K, Ca, etc), transport ATPases, metabolism of secondary products derived from L-lysine, L-arginine, and L-histidine, and endocytosis. Note that some proteins are included in more than one functional category because FunSpec does not compensate for proteins in multiple categories.

**Table 1:** Functional categories overrepresented with proteins whose absence renders cells more sensitive to NiSO_4_.

MIPS Functional Category	p-value	In Category from Cluster	# Nickel Toxicity Modulating	Total in Category
homeostasis of proteins [34.01.01.03]	0.000556	VMA2 CUP5 RAV1 VMA6 STV1 VPH1	6	47
cation transport (H+, Na+, K+, Ca2+, NH4+, etc.) [20.01.01.01]	0.0007315	VMA2 CUP5 TOK1 MNR2 VMA6 STV1 VPH1	7	68
siderophone-iron transport [20.01.01.01.01.01]	0.002052	FTH1 AFT1 FET3	3	12
homeostasis of metal ions (Na, K, Ca etc.) [34.01.01.01]	0.006003	FTH1 CUP5 AFT1 TOK1 MNR2 MAC1 FET3	7	98
transport ATPases [20.03.22]	0.00621	VMA2 CUP5 VMA6 STV1 VPH1	5	53
metabolism of secondary products derived from L-lysine, L-arginine and L-histidine [01.20.31]	0.006963	JJJ3DPH2	2	6
endocytosis [20.09.18.09.01]	0.009743	FTH1 RVS161 CUP5 YPK1 WHI2	5	59

#### Homeostasis of protons, cation transport, transport ATPases, and endocytosis

As is expected for cells treated with agents that are actively internalized by the cell a number of deletion strains of proteins involved in endocytosis exhibited sensitivity to NiSO_4 _(Fth1, Rvs161, Cup5, Ypk1, Whi2) (Table 1). Proteins in the proton homeostasis, cation transport, and transport ATPases MIPS functional categories include subunits of the vacuolar-ATPase, Vma2p, Cup5p, Vma6p, Stv1p, and Vph1p (Table 1). Vacuolar ATPases (V-ATPases) are multi-subunit ATP-dependent proton pumps that play an important role in the pH homeostasis of various intracellular compartments and allow cellular processes such as autophagy, endocytosis and intracellular transport to take place. It is not surprising that gene deletion of proteins that function in vacuolar processes renders cells more sensitive to nickel compounds since genes involved in vacuolar organization and biogenesis have been shown essential for the cell's viability in response to metal exposure [30-32]. The vacuolar pH gradient-driven system allows the penetration of nickel ions into vacuoles and the formation of histidine-nickel ion complexes sequester excess amounts of nickel ions into vacuoles [31,33,34]. Sequestering metals into vacuoles may be a fundamental process for *S. cerevisiae *in mediating resistance to metal toxicity.

#### Siderophore-iron transport and homeostasis of metal ions

The siderophore-iron transport MIPS functional category includes Fth1p, a putative high affinity iron transporter, Aft1p, a transcription factor involved in activating the expression of target genes in response to cellular changes in iron availability, and Fet3p, required for high affinity iron uptake (Table 1). Nickel compounds have been shown to interfere with iron uptake, deplete cellular iron levels, and interfere with the function of enzymes that require iron for their enzymatic activity [16,26]. Toxic metal-induced iron depletion may be a common feature of many toxic metals [31]. Because nickel ions, structurally or chemically, resemble essential metal ions such as zinc, copper, iron, and manganese, Ni^+2 ^could compete with and displace these metal ions from the cell [22]. Therefore, the sensitivity to nickel exhibited by deletion strains of proteins involved in metal ion homeostasis, such as, the putative magnesium transporter Mnr2p and the Mac1 protein, a transcription factor involved in regulation of genes required for high affinity copper transport, is not surprising (Table 1).

#### Metabolism of secondary products derived from L-lysine, L-arginine, and L-histidine

**Figure 2:**
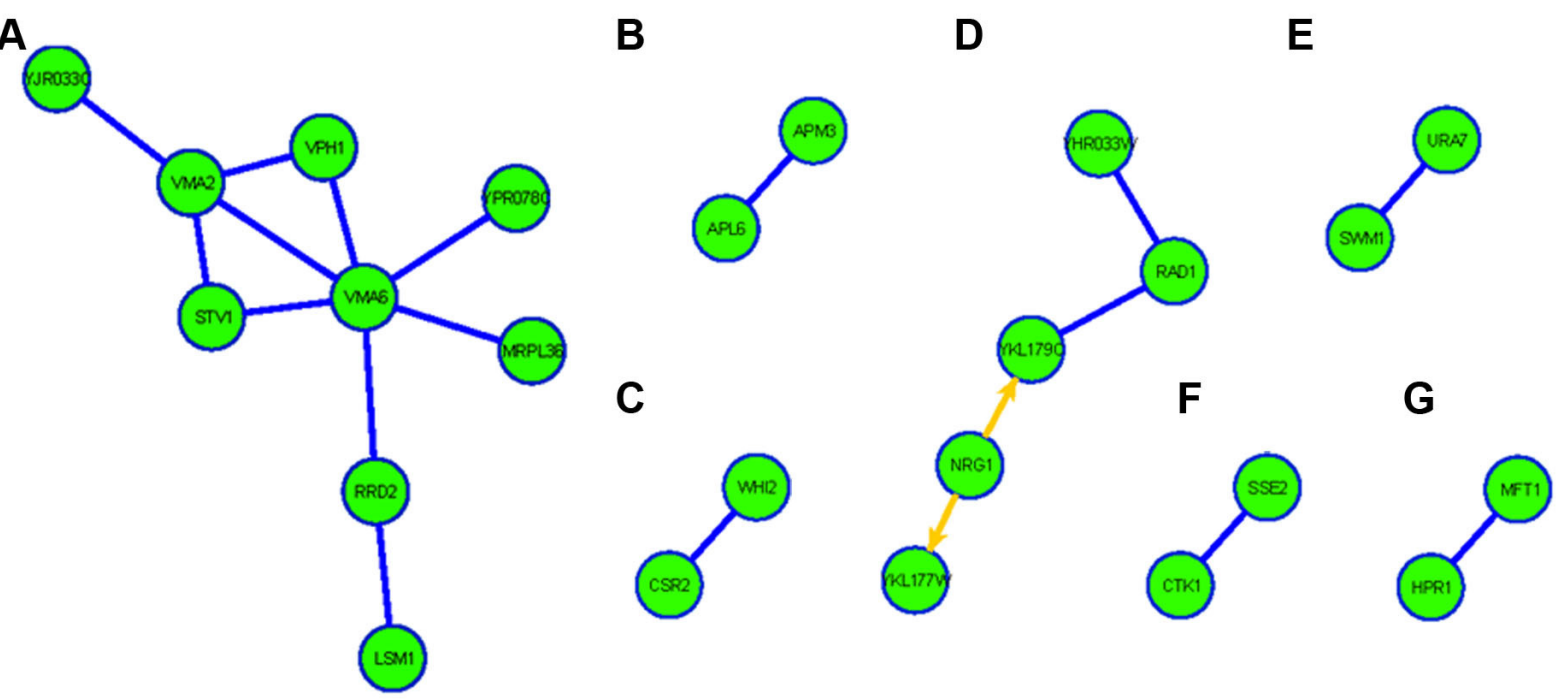
**Nickel toxicity modulating networks identified with proteins whose absence renders cells sensitive to NiSO_4_**. The yeast protein interactome consisting of 5,433 proteins, 14,656 protein-protein interactions, and 5,621 protein-DNA interactions was compiled using the program Cytoscape. Proteins corresponding to nickel sensitive gene deletion strains were mapped onto the interactome and then filtered to identify connected groups of proteins (N => 2). Straight lines indicate protein-protein interactions and arrows indicate DNA-protein interaction.

The MIPS functional category metabolism of secondary products derived from L-lysine, L-arginine, and L-histidine includes the Jjj3 and Dph2 proteins (Table 1). Both these proteins play an essential role in the biosynthesis of diphthamide (DPH), an unusual amino acid formed by the posttranslational modification of a conserved histidine found in the translation elongation factor, eEF2 [35]. This modified amino acid is the target for ADP-ribosylation by the diphtheria toxin (DT). As a result, cells lacking Jjj3 or other proteins involved in this pathway are tolerant to DT. Although evolutionarily conserved, the biological function of diphthamide within the activity of eE2F remains elusive. Recent work has shown a requirement for diphthamide in the maintenance of translational fidelity by maintaining the correct reading frame during translocation across the ribosome [36]. To our knowledge, the diphthamide biosynthesis pathway has not been previously linked to nickel toxicity. Interestingly, the Jjj3 protein encompasses a J domain (region with homology to the E. coli DnaJ protein). The J domain is characterized by a highly conserved histidine-proline-aspartic acid (HPD) tripeptide signature motif important for stimulation of the ATPase activity of their Hsp70 partner [37]. Another possibility is that its activity as chaperone to hsp90 confers the Jjj3 deletion strain sensitive to NiSO_4_. Previous evidence does suggest that induction of stress proteins may play a role in the cellular response to heavy metal exposure [38,39]. Our screen also identified the deletion strain of Sse2p, a member of the heat shock protein 70 (HSP70) family, as sensitive to nickel (figure 2f).

### Protein interactome analysis with proteins whose absence renders cells sensitive to NiSO_4_

The first nickel toxicity modulating network identified includes the interaction between Rav1 (Yjr033C), a subunit of the RAVE complex, that promotes assembly of the V-ATPase holoenzyme (Vma2, Vph1, Stv1, Vma6) (figure 2a). Vma6 interacts with Ypr078C, a protein with a possible role in DNA metabolism and/or in genome stability, Mrpl36, a mitochondrial ribosomal protein, and Rrd2, an activator of the phosphotyrosyl phosphatase activity of Pp2a, a peptidyl-prolyl cis/trans-isomerase that regulates G1 phase progression, the osmoresponse, and microtubule dynamics. Rrd2 also interacts with Lsm1, a protein involved in the degradation of cytoplasmic mRNAs. Also associated with the activation of the stress response is Whi2p. A nickel toxicity modulating network was identified between Whi2p and Csr2, a nuclear protein proposed to regulate utilization of nonfermentable carbon sources and endocytosis of plasma membrane proteins (figure 2c).

Deletion strains of Apl6p and Apm3p, subunits of the yeast alkaline phosphatase pathway (AP-3 complex) that functions in protein transport from the Golgi directly to the vacuole without proceeding through an endosome intermediate, exhibited nickel-sensitivity (figure 2b). Deletion strains of components of the AP-3 complex have been shown to be specifically associated with cellular sensitivity to nickel [32]. It is unclear why gene deletion of components of the alkaline phosphatase pathway renders cells sensitive to nickel while deletion of components of other transport pathways to the vacuole results in nickel-resistance (discussed below).

The fourth nickel toxicity modulating network includes the protein-DNA interaction between Nrg1 and Coy1 (Ykl179C) (figure 2d). Nrg1 is a transcriptional repressor that mediates glucose repression and negatively regulates a variety of processes including filamentous growth and the alkaline pH response [40-43]. Coy1 (Ykl179C) is a Golgi membrane protein, with similarity to mammalian Casp, with a suggested role in intra-Golgi retrograde transport. Coy1 physically interacts with Rad1, a single-stranded DNA endonuclease subunit of Nucleotide Excision Repair Factor 1 (NEF1), homolog of human XP. RAD1 interacts with Yhr033W, a putative protein of unknown function. Because the biological function of both Coy1 and Yhr033W is not well understood, the biological relevance of their interaction with Rad1 cannot be explained at the moment. Interestingly, Rim101, whose gene deletion strain is resistant to nickel (Table 2), has been recently implicated in the control of cell wall assembly and is a direct transcriptional repressor of Nrg1 [41]. Rim101 governs pH-dependent responses by repressing Nrg1, and Nrg1p negatively regulates alkaline pH-induced genes [41]. The deletion strain of Rim101 exhibits reduced nickel ion accumulation levels and is also resistant to cadmium [31]. There is also a protein-DNA interaction between Nrg1p and Ykl177W, a dubious open reading frame.

**Table 2:** Functional categories overrepresented with proteins whose absence renders cells more resistant to NiSO_4_.

MIPS Functional Category	p-value	In Category from Cluster	# Nickel Toxoicity Modulating	Total in Category
vacuolar/lysosomal transport [20.09.13]	<1e-14	VPS8 BSD2 STP22 VPS64 PEP7 VPS3 VPS29 VPS35 VPS25 SNF7 VTA1 VPS38 VPS36 VPS20 VPS75 VPS27 TLG2 VMA4 VTS1 SNF8 VPS28 BRO1 VPS30	23	153
protein targeting, sorting and translocation [14.04]	2.18e-13	VPS8 SEC66 BSD2 STP22 VPS64 PEP7 VPS3 GOS1 VPS29 PEP8 VPS35 VPS25 SNF7 VPS38 VPS36 VPS75 VPS27 TLG2 RTG1 VPS5 VPS17 SNX3 VTS1 SNF8 VPS28 VPS30 TRE1	27	261
intra Golgi transport [20.09.07.05]	2.039e-05	GOS1 PEP8 VPS35 VPS36 VPS27 VPS5	6	33
regulation of C-compound and carbohydrate metabolishm [01.05.25]	7.105e-05	TPS1 REG1 NGG1 SSN2 RTG2 VPS25 SNF7 VPS36 RTG1 SNF8	10	126
vacuole or lysosome[42.25|	0.0001109	KCS1 DOA4 VTC1 VPS29 TLG2 VAM10	6	44
transcription, repression [11.02.03.04.03]	0.0001137	RIM101 VPS25 VPS36 SFL1 SNF8	5	28
chromosome segregation/division [10.03.04.05]	0.003745	IML3 CHL4 MCM21 NNF2 CTF19	5	59
vesicular transport (Golgi network, etc.) [20.09.07]	0.008728	PMR1 VPS29 VPS17 VPS30 APL5	5	72

A nickel toxicity modulating network was identified between Ura7p and Swm1p (figure 2e) and the interaction between Ctk1p and Sse2p (figure 2f). Ura7p is involved in the final step in the *de novo *biosynthesis of pyrimidines and Swmp1 is a subunit of the anaphase-promoting complex, an E3 ubiquitin ligase that regulates the metaphase-anaphase transition and exit from mitosis. Ctk1p is the catalytic alpha subunit of the C-terminal domain Kinase I (CTDK-1) involved in transcription and pre-MNA 3'end processing, and translational fidelity and Sse2p is a member of the heat shock protein 70 (hsp70) family.

The last nickel toxicity modulating network is the interaction between Hpr1 and Mft1 (figure 2g), components of the evolutionarily conserved THO nuclear complex, present in a larger complex, termed, TREX, and with components of the nuclear export machinery [44-48]. The THO/TREX complex is functionally involved in mRNP biogenesis and transport, required for transcriptional elongation, and is a key player in the coupling of transcription and RNA export [44-48]. Our finding that members of the THO complex play a role in the toxicity of yeast cells to NiSO_4 _is supported by a recent finding that described the sensitivity of deletion strains of proteins involved in nucleocytoplasmic transport (including pore complex formation, and functionality) to both nickel and cadmium [32]. The deletion strain of Mft1, a subunit of the THO nuclear complex, was identified as the most sensitive strain to nickel in our secondary validation screen and the deletion strain of Hpr1 was identified as the sixteenth most sensitive strain (Additional file 2). The exact role that the nucleocytoplasmic transport process, more specifically the THO complex, plays in nickel toxicity still needs to studied.

### Functional categories overrepresented and nickel resistance networks identified with proteins whose absence renders cells resistant to NiSO_4_

Functional categories overrepresented with proteins whose absence renders cells resistant to nickel were identified using FunSpec (Table 2).

#### Vacuolar/lysosomal transport and function

**Figure 3:**
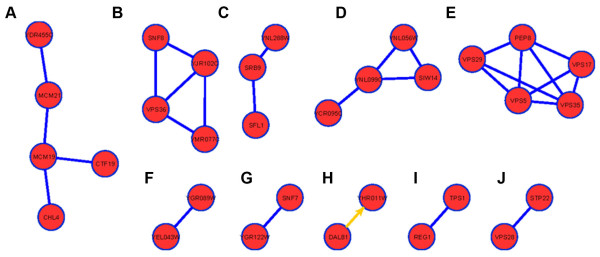
**Nickel resistance networks identified with proteins whose absence renders cells resistant to NiSO_4_**. The yeast protein interactome consisting of 5,433 proteins, 14,656 protein-protein interactions, and 5,621 protein-DNA interactions was compiled using the program Cytoscape. Proteins corresponding to nickel resistant gene deletion strains were mapped onto the interactome and then filtered to identify connected groups of proteins (N => 2). Straight lines indicate protein-protein interactions and arrows indicate DNA-protein interaction.

A number of deletion strains whose gene deletion product is a component of the multivesicular body sorting (MVB) pathway were found resistant in our screen. The MVB sorting pathway provides a mechanism for lysosomal degradation of transmembrane proteins and plays a critical role in a diverse range of processes, including growth factor receptor down-regulation, antigen presentation, developmental signaling, and the budding of enveloped viruses. Three high molecular weight cytoplasmic complexes function in MVB sorting, the endosomal sorting complexes (ESCRT) I, II, and III. The ESCRT-I complex (Vps23, Vps28, and Vps37) recruited to endosomes by Vps27, interacts with ubiquitinated proteins and initiates the MVB sorting reaction [49]. The ESCRT-II complex (Snf8, Vps36, and Vps25) functions downstream of ESCRT-I [50] and then recruits the ESCRT-III subunits (Snf7, Vps20, Vps2, and Vps24) to the endosome, where they oligomerize to form the ESCRT-III complex [51]. ESCRT-III recruits accessory factors such as Bro1, which in turn recruits Doa4 [52,53], the deubiquitinating enzyme that removes ubiquitin from MVB target proteins before their sorting into MVB vesicles. ESCRT-III also recruits the AAA-type ATPase Vps4 that catalyzes the disassembly of the ESCRT machinery and recycles the ESCRT complexes into the cytosol to allow further rounds of target protein sorting [54]. The importance of the MVB sorting pathway in the toxicity of *S. cerevisiae *to nickel compounds is evident by the fact that many deletion strains of members of this pathway were found resistant to nickel in our screen. For example, members of the ESCRT complexes I (Vps28), and Stp22 that interacts with Vps28, II (Snf8, Vps36, Vps25), and III (Snf7, Vps20) were found resistant to NiSO_4 _(Table 2). Deletion strains of Bro1 and the ubiquitinating enzyme Doa4 were also found resistant, as well as, Vta1, a protein in the MVB pathway that regulates the activity of Vps4. Additionally, two nickel resistance networks were identified in the MVB pathway (figure 3b and figure 3g). The relationship between the toxicity of yeast cells and the resistance of deletion strains of the MVB pathway to nickel compounds is not clear. However, recently it was shown that deletion strains of the MVB pathway (including Bro1 and Snf8) exhibited resistance to NiCl_2 _and a reduction in intracellular nickel ion accumulation levels suggesting that export and or reduced uptake may underlie the nickel resistance displayed by these mutant strains [32,55].

#### Targeting, sorting, translocation of proteins and intra-Golgi transport

The gene product of deletion strains resistant to nickel also included proteins targeted, sorted and translocated to the Golgi (Table 2). For example, the Vps29, Vps35, Vps5, Vps17 and Pep8 multimeric membrane-associated retromer complex essential for endosome-to-Golgi retrograde protein transport was identified as one of the nickel resistance networks (figure 3e). Also involved in endosome-to-Golgi protein transport is Snx3p, a sorting nexin, Vps27, an endosomal protein required for recycling Golgi proteins, components of t-SNARE (Tlg2p), v-SNARE (Vts1p and Gos1p) and Pmr1, a high affinity Ca^2+^/Mn^2+ ^P-type ATPase required for Ca^2+ ^and Mn^2+ ^transport into the Golgi (Table 2). The deletion strain of Pep7p, essential for targeting of vesicles to the endosome and required for vacuole inheritance (Golgi to vacuole transport), components of the CORVET complex (Vps8p and Vps3p), required for localization and trafficking of the CPY sorting receptor from late endosome to vacuole, and Vps38, that functions in carboxypeptidase Y (CPY) sorting were also found resistant to nickel (Table 3). The last nickel resistance network indentified is the interaction between Vps8p and Stp22p (figure 3j). Vps8 is a component of the CORVET complex required for CPY sorting receptor from late endosome to vacuole and Stp22p is a component of the ESCRT-1 complex in the MVB pathway (discussed above). The interaction between Vps8p and Stp22p further validates our results of the involvement of components of the MVB pathway and endosome to vacuole transport in mediating the toxicity *S. cerevisiae *cells to nickel compounds.

#### Transcriptional repression and regulation of C-compound and carbohydrate metabolism

The MIPS functional category, transcription repression, includes Rim101p, Vps25p, Vps36p, Sfl1p, and Snf8p. Rim101 is involved in cell wall construction and cellular response to pH changes (discussed above) (Table 2) [41]. Vps25, Vps36, and Snf8, components of the ESCRT-II complex, are also involved in derepressing the expression of glucose repressed genes. The ESCRT-II complex subunits are the yeast orthologues of the human RNA polymerase II elongation factor ELL associated proteins (EAPs) that together with ELL form a 'holo-ELL complex' that increases the catalytic rate of transcription elongation by RNA polymerase II *in vitro *[56]. The possibility that the ESCRT-II complex has acquired a nuclear function in mammalian cells and lost its importance in membrane trafficking has been postulated since it's believed that these subunits might be dispensable for MVB sorting in mammals [56,57]. Also resistant in our screen is the Sfl1 transcriptional repressor and activator of stress response genes as well as the nickel resistance interaction network involved in transcriptional regulation between Ynl288Wp, Srb9p, and Sfl1p and the interaction between Reg1p and Tps1p involved in carbohydrate metabolism and stress response (figure 3c and figure 3i).

#### Chromosome segregation and division

Interestingly, components of the chromosome segregation/division MIPS functional category were found overrepresented in our list of nickel-resistant strains (Table 2, figure 3a). These include: Ydr455Cp, Mcm21p, Mcm19p, Chl4p, and Ctf19p, subunits of the outer kinetochore required for choromosome stability that provide a link between centromere DNA binding proteins of the inner kinetochore and microtubule-binding proteins. To the best of our knowledge, the subunits of the outer kinetochore have not been previously linked to nickel toxicity. Additionally, a nickel resistance network was found between Ygr089W, involved in chromosome segregation, and Yel043W, a cytoskeletal protein (figure 3f).

Other nickel resistance networks identified include the interaction between Ynl056W, Ynl099C, Siw14, and Ycr095C, that plays a role in cell cycle arrest in response to oxidative DNA damage (figure 3d), and the interaction between Dal81, a positive regulator of genes in multiple nitrogen degradation pathways, and Yhr011W, a probable mitochondrial seryl-tRNA synthetase (figure 3h).

### Functional categories overrepresented with proteins that have a similar human protein whose gene deletion renders cells sensitive or resistant to NiSO_4_

We further restricted our list of proteins whose absence renders cells sensitive or resistant to nickel to only those proteins that have a similar protein in human cells. A protein in human cells similar in amino acid sequence to the yeast protein was identified for 68% of the proteins whose gene deletion cause sensitivity or resistance to NiSO_4_. Note that only the top scoring human protein was used. This analysis identified 111 nickel-sensitive and 72 resistant strains. To further study the degree of sensitivity or resistance of each deletion strain in our phenotypic screen, the IC_50 _for each deletion strain was analyzed in a secondary validation screen and the target strains were arranged based on their degree of sensitivity to NiSO_4_. Sensitivity increased with decreasing IC_50 _and resistance increased with increased IC_50_. A list of proteins corresponding to the nickel sensitive and resistant strains identified in our screen including yeast systematic number, symbol, function, similar human protein and IC_50 _is included in Additional file 2.

Functional categories overrepresented in the list of nickel-sensitive and resistant deletion strains whose gene deletion product has a similar human protein are provided in Additional files 3 and 4. The five nickel toxicity modulating and four nickel-resistance networks are included in Additional files 5 and 6. The cell cycle MIPS functional category emerged as a category not previously identified in our initial analysis of yeast proteins with and without similar human proteins (Table 1). This category includes the Pin4, Far7, and Far10 proteins (Additional file 3). Pin4p, containing an RNA recognition motif, is involved in normal G2/M cell cycle progression and is hyperphosphorylated in response to DNA damage [58]. Its human homologue, the cleavage stimulation factor 64 kDa subunit, tau variant, is also an RNA-binding protein phosphorylated upon DNA damage [59,60]. Far7p and Far10p interact and have been shown to be involved in G1 cell cycle arrest in response to pheromone [61]. The human homologue of Far7, tpr, is involved in protein import into the nucleus and is also phosphorylated upon DNA damage [60,62]. The human homologue of Far10, the Centromere protein F (CENP-F), is involved in chromosome segregation during mitosis; CENP-F is hyperphosphorylated during mitosis and upon DNA damage, and gradually accumulated during the cell cycle [60,63]. In general, checkpoints control the ability of cells to arrest in a specific phase of the cell cycle in response to DNA damage or other stresses, and allow the cell enough time to recruit and activate repair machineries, and to repair the damage before passing to the next cell cycle phase. Although Ni (II) is a weak DNA-damaging agent, it has been shown to interfere with nucleotide and base excision repair at low noncytotoxic concentrations [64]. Additionaly, nickel-induced effects on the cell cycle have been previously reported [64-66]. Analysis of the cell-cycle effect of a 24 h exposure to 1 mM NiCl_2 _in A549 cells indicated a loss in the amount of cells in S phase and a corresponding increase in the percentage of cells in G_1 _but no significant change in cells in G_2_/M [65,66]. Because impairment of protective mechanisms by nickel compounds and other toxic metals may lead to increased toxicity and increased risk of carcinogenesis, future investigations should focus on the mechanism(s) by which nickel induces G1 cell cycle arrest, for example, by inducing DNA damage or by inhibiting DNA damage repair activity, or by both. As *S. cerevisiae *has proven to be an excellent model organism, and one in which parallel processes with homologous gene products can be determined in mammalian cells, future studies will examine the role that these human homologues may play in regulating toxicity to nickel in human cells. Additionally, we will examine if the pathways found overrepresented with those proteins whose absence renders cells more sensitive or resistant to nickel are also affected in human cells in response to nickel exposure.

## Conclusion

Genomic phenotypic screens have been useful in the past to determine the role of nonessential proteins in modulating toxicity after exposure to an environmental agent [1,5-10]. Here, we have screened a library of *S. cerevisiae *single-gene deletion mutant strains corresponding to the complete set of 4733 nonessential yeast genes with NiSO_4 _to identify those strains most sensitive or resistant to nickel. We have identified 149 genes whose gene deletion causes sensitivity to NiSO_4 _and 119 genes whose gene deletion confers resistance. Pathway analysis with the list of proteins whose gene deletion causes sensitivity and resistance to nickel identified a wide range of cellular processes engaged in the toxicity of *S. cerevisiae *to NiSO_4_. Functional categories overrepresented with proteins whose absence renders cells sensitive to NiSO_4 _include homeostasis of protons, cation transport, transport ATPases, endocytosis, siderophore-iron transport, homeostasis of metal ions, and the diphthamide biosynthesis pathway. Functional categories overrepresented with proteins whose absence renders cells resistant to nickel include functioning and transport of the vacuole and lysosome, protein targeting, sorting, and translocation, intra-Golgi transport, regulation of C-compound and carbohydrate metabolism, transcriptional repression, and chromosome segregation/division. Seven nickel toxicity modulating networks and ten nickel resistance networks were identified. The biological function of the nickel toxicity modulating networks and resistance networks also highlight the pathways described above as well as identify components of the alkaline phosphatase pathway and THO nuclear complex as mediating sensitivity to nickel. Additionally, we studied the degree of sensitivity or resistance to nickel of the 111 nickel-sensitive and 72 resistant strains whose gene deletion product has a similar protein in human cells.

Several genome-wide phenotypic screens have examined the toxicity of *S. cerevisiae *to metal compounds [30,32,55,67,68]. To date, one study has reported data from a genomic phenotypic screen using NiCl_2 _and another study screened a *S. cerevisiae *library with NiCl_2 _and measured the intracellular Ni ion levels of each deletion strain in the library [32,55]. We found that 15% of our sensitive strains and 31% of our resistant strains overlapped with those identified by Ruotolo *et al*. and 9% of our sensitive and 13% of our resistant strains overlapped with those identified by Eide *et al*. [32,55]. The small overlap found between our study and that of Ruotolo *et al*. and Eide *et al*. could be due to the differences in the background of the strains used as well as the fact that while our phenotypic screen was carried out using NiSO_4_, both these screens were carried out using NiCl_2_. Also worthy of note is that while our phenotypic study screened for growth advantage and disadvantage under NiSO_4 _exposure the Eide *et al*. identified gene deletion strains whose intracellular Ni ion levels differed from the parental strain but whose growth may or may not have been affected by nickel exposure. In the past the overlap between data of two different phenotypic screens has been between 10-20% since screens are usually carried out in dissimilar conditions and the sensitivity or resistance of strains to a specific agent may be determined differently [69]. Apart from the small overlap in strains found between our study and that of Routolo *et al*., many of the same pathways were found overrepresented in both studies. Ruotolo *et al*. also reported a sensitivity to nickel exhibited by deletion strains whose gene product is involved in the V-ATPase and endocytosis, Golgi-to-vacuole transport, nucleocytoplasmic transport, and the alkaline phosphatase pathway and a resistance to nickel by strains whose gene product is involved in the MVB pathway, endosome transport, and endosome-to-Golgi transport retrograde transport [32].

Data from phenotypic screens with metals has identified several common pathways that modulate metal tolerance in *S. cerevisiae*. As is the case with nickel, deletion strains of V-ATPase subunits and vacuolar transport and function have been found sensitive to cadmium, mercury, arsenite, cobalt, zinc, and iron [32]. The vacuole appears to be a hot spot for metal toxicity since vacuolar transport allows metal sequestration in the vacuole preventing damage to the cell and may be important for processing and trafficking of response proteins and removing damaged proteins. Nucleocytoplasmic transport, iron transport, and Golgi-to-vacuole transport may also be hot spots for metal toxicity since deletion strains of proteins involved in these pathways were also found sensitive to cadmium [32]. Chelating metals, sequestering metals into vacuoles, and reducing cellular stress are fundamental processes for mediating resistance to metals by *S. cerevisiae *[30].

Metal-specific responses in mediating toxicity of *S. cerevisiae *to an exogenous agent have also been reported [30,32,55,67,68]. For example, mutants sensitive to nickel are significantly enriched in stress-related transcription regulation, tubulin folding, signal transduction, the secretory pathway and response to stimulus while mutants sensitive to cadmium are enriched in cell surface receptor linked signal transduction, morphogenesis, chromatin modification, glutathione biosynthesis and DNA damage [30]. A metal-specific response identified in both our study and that of Ruotolo *et al*. is the resistance of deletion strains of components of the MVB pathway (ESCRT complexes) to nickel. It is unclear at the moment why deletion of components of the ESCRT complex render cells resistant to nickel but sensitive to other metals such as cobalt and cadmium [32]. Another nickel-specific response identified in both the Ruotolo *et al*. study and our study is the sensitivity of deletion strains of components of the alkaline phosphatase pathway. It is also unknown why deletion of components of the alkaline phosphatase pathway renders cells sensitive to nickel while deletion of components of other transport pathways to the vacuole results in nickel-resistance. However, our most interesting findings are the increased sensitivity of deletion strains of components of the diphthamide biosynthesis pathway to nickel and the resistance of deletion strains of proteins involved in chromosome segregation and division. The results of this study suggest a possible role of the evolutionarily conserved diphthamide biosynthesis pathway as well as components of the outer kinetochore involved in chromosome segregation in mediating nickel toxicity in *S. cerevisiae*.

We have undertaken a whole genome approach in order to further understand the mechanism(s) regulating the cell's toxicity to nickel compounds and have used computational methods to integrate the data and generate global models of the yeast's cellular response to NiSO_4_. Future studies will focus on determining if the gene product of the nickel sensitive and resistant strains regulates the level of nickel ions within the cell. We would also like to determine if the same pathways identified in mediating toxicity of *S. cerevisiae *to nickel also play a role in regulating the toxicity of human cells to nickel compounds.

## Methods

### *S. cerevisiae *genomic phenotypic screen with NiSO_4_

Genomic phenotyping with the *S. cerevisiae *strain BY4741 and single-gene deletion mutant derivatives corresponding to the nonessential yeast genes was performed as previously described [1,5]. Briefly, 96-well master plates containing individual deletion strains were grown in 150 μl of YPD (10 g yeast extract, 20 g peptone, 20 g dextrose, 20 g agar/liter), containing G418 at 200 μg/ml. Settled cells in each position of the 96-well plate were resuspended with 60 μl bursts of forced air from a Hydra liquid handling apparatus (Robbins Scientific), and then using the Hydra, 1 μl samples were spotted simultaneously onto an agar-containing plate. NiSO_4 _was purchased from Sigma. Plates containing up to 96 strains were tested under the following conditions: no treatment, 0.75 mM (low concentration) and 1.25 mM (high concentration) NiSO_4_. Strains were grown for 60 hrs at 30°C and then imaged with AlphaImager software (Alpha Innotech Corporation, San Leandro, CA). The screen was performed in triplicate with fresh liquid cultures. Sensitive and resistant strains were identified by visual inspection of the images. Strains were labeled sensitive to NiSO_4 _if nickel treatment inhibited its growth. Strains were labeled resistant if nickel treatment did not inhibit its growth. The single gene deletion of random strains was verified using a standard genomic DNA PCR protocol with primers flanking 100 bp upstream of the transcriptional start site and 100 bp downstream of the stop site of the specific gene knocked out. This confirmed that strains in the library contain a single gene deletion knockout and had not been contaminated with other strains.

### Secondary screen

A secondary screen of those strains identified in the primary screen whose gene deletion product have a protein in human cells similar in amino acid sequence was performed by calculating the Inhibitory Concentration (IC_50_) of each individual deletion strain to NiSO_4 _using the Graph Pad Prism 5 software. Briefly, 96 well plates containing individual deletion strains were grown in 200 μl of YPD media containing G418 at 200 μg/ml and increasing doses of NiSO_4_. The concentrations of NiSO_4 _used in the secondary screen were: no treatment, 0.375 mM, 0.75 mM, 1.0 mM, 1.25 mM, and 2.5 mM NiSO_4_. The cultures were incubated at 30°C for 20 h. Growth of each strain after treatment was monitored by measuring the cell density at 590 nM using the Perkin Elmer HTS 7000 Bio Assay Reader. The IC_50 _is defined as the concentration of NiSO_4 _that inhibits 50% of the growth of each individual strain compared to the growth of that strain under no treatment. Similar proteins between *S. cerevisiae *and humans were identified using the BLAST program available from the National Center for Biotechnology Information [70]. The *S. cerevisiae *protein sequence was used to query the translated nucleotide database specific to humans. Note only the top scoring human protein was used.

### Data analysis

The program FunSpec was used to identify those functional categories overrepresented with our list of proteins whose *absence *renders cells sensitive or resistant to nickel. Our list of the gene deletion products sensitive and resistant to nickel were input into FunSpec, and FunSpec, based on prior knowledge, integrates the data and provided a summary of the MIPS functional categories overrepresented in the list [71]. The *p*-values in FunSpec represent the probability that the intersection of a given list with any functional category occurs by chance. Interactome analysis was done using the program Cytoscape. *S. cerevisiae *protein interaction information was downloaded from the Database of Interacting Proteins (DIP). Protein-DNA interactions were derived from a previously published study [72]. Protein-protein interaction information was imported into Cytoscape for network visualization and subnetwork filtering. Filtering was performed by highlighting Ni-toxicity modulating proteins and their associated protein-protein and protein-DNA interactions [73-75].

## Authors' contributions

AA carried out the genomic phenotypic screen, secondary validation screen, wrote the manuscript. XZ carried out the genomic phenotypic screen and secondary validation screen. TPE carried out the genomic phenotypic screen. XL carried out the genomic phenotypic screen JB carried out the genomic phenotypic screen. JPR provided technical support to the phenotypic screen and helped out with the phenotypic screen. AK helped analyze the results of the phenotypic screen. TJB provided conception and design, or acquisition of data, or analysis and interpretation of data and critically revised the manuscript. MC provided conception and design, or acquisition of data, or analysis and interpretation of data, critically revised the manuscript and has given final approval of the version to be published. All authors have read and approved the manuscript.

## Supplementary Material

Additional file 1 **Proteins corresponding to the gene product of the *S. cerevisiae *NiSO_4_-sensitive and resistant gene deletion strains**. The table includes the yeast systematic number of the 149 *S. cerevisiae *proteins whose gene deletion causes sensitivity to NiSO_4 _and 119 proteins whose gene deletion causes resistance. Click here for file

Additional file 2 **Proteins corresponding to the gene product of the *S. cerevisiae *NiSO_4_-sensitive and resistant gene deletion strains**. The table includes the yeast systematic number, symbol, function, and human homologue to the protein corresponding to the gene product of NiSO_4 _sensitive and resistant gene deletion strains identified in our screen. The list is arranged from lowest to highest IC_50_. Sensitivity to NiSO_4 _increases with decreasing IC_50 _and resistance increases with increasing IC_50_. Click here for file

Additional file 3 **Functional categories overrepresented with proteins (that have a similar human protein) whose absence renders cells more sensitive to NiSO_4_**. Functional categories overrepresented with proteins whose absence renders cells sensitive to nickel were identified using FunSpec. Click here for file

Additional file 4 **Functional categories overrepresented with proteins (that have a similar human protein) whose absence renders cells more resistant to NiSO_4_**. Functional categories overrepresented with proteins whose absence renders cells resistant to nickel were identified using FunSpec. Click here for file

Additional file 5 **Nickel toxicity modulating networks identified with proteins (that have a similar human protein) whose absence renders cells more sensitive to NiSO_4_**. The yeast protein interactome consisting of 5,433 proteins, 14,656 protein-protein interactions, and 5,621 protein-DNA interactions was compiled using the program Cytoscape. Proteins corresponding to nickel sensitive gene deletion strains were mapped onto the interactome and then filtered to identify connected groups of proteins (N => 2). Straight lines indicate protein-protein interactions. Click here for file

Additional file 6 **Nickel resistance networks identified with proteins (that have a similar human protein) whose absence renders cells more resistant to NiSO_4_**. The yeast protein interactome consisting of 5,433 proteins, 14,656 protein-protein interactions, and 5,621 protein-DNA interactions was compiled using the program Cytoscape. Proteins corresponding to nickel resistant gene deletion strains were mapped onto the interactome and then filtered to identify connected groups of proteins (N => 2). Straight lines indicate protein-protein interactions and arrows indicate DNA-protein interaction. Click here for file

## References

[B1] Begley TJ Rosenbach AS Ideker T Samson LD Hot spots for modulating toxicity identified by genomic phenotyping and localization mapping Mol Cell 2004 16 1 117 125 10.1016/j.molcel.2004.09.005 15469827

[B2] Goffeau A Barrell BG Bussey H Davis RW Dujon B Feldmann H Galibert F Hoheisel JD Jacq C Johnston M Life with 6000 genes Science 1996 274 5287 563 547 10.1126/science.274.5287.546 8849441

[B3] Johnston M The yeast genome: on the road to the Golden Age Curr Opin Genet Dev 2000 10 6 617 623 10.1016/S0959-437X(00)00145-3 11088011

[B4] Winzeler EA Shoemaker DD Astromoff A Liang H Anderson K Andre B Bangham R Benito R Boeke JD Bussey H Functional characterization of the S. cerevisiae genome by gene deletion and parallel analysis Science 1999 285 5429 901 906 10.1126/science.285.5429.901 10436161

[B5] Begley TJ Rosenbach AS Ideker T Samson LD Damage recovery pathways in Saccharomyces cerevisiae revealed by genomic phenotyping and interactome mapping Mol Cancer Res 2002 1 2 103 112 12496357

[B6] Bennett CB Lewis LK Karthikeyan G Lobachev KS Jin YH Sterling JF Snipe JR Resnick MA Genes required for ionizing radiation resistance in yeast Nat Genet 2001 29 4 426 434 10.1038/ng778 11726929

[B7] Birrell GW Giaever G Chu AM Davis RW Brown JM A genome-wide screen in Saccharomyces cerevisiae for genes affecting UV radiation sensitivity Proc Natl Acad Sci USA 2001 98 22 12608 12613 10.1073/pnas.231366398 11606770 PMC60101

[B8] Game JC Birrell GW Brown JA Shibata T Baccari C Chu AM Williamson MS Brown JM Use of a genome-wide approach to identify new genes that control resistance of Saccharomyces cerevisiae to ionizing radiation Radiat Res 2003 160 1 14 24 10.1667/RR3019 12816519

[B9] Stepchenkova EI Kozmin SG Alenin VV Pavlov YI Genome-wide screening for genes whose deletions confer sensitivity to mutagenic purine base analogs in yeast BMC Genet 2005 6 1 31 10.1186/1471-2156-6-31 15932646 PMC1173102

[B10] Wu HI Brown JA Dorie MJ Lazzeroni L Brown JM Genome-wide identification of genes conferring resistance to the anticancer agents cisplatin, oxaliplatin, and mitomycin C Cancer Res 2004 64 11 3940 3948 10.1158/0008-5472.CAN-03-3113 15173006

[B11] Salnikow K Zhitkovich A Genetic and epigenetic mechanisms in metal carcinogenesis and cocarcinogenesis: nickel, arsenic, and chromium Chem Res Toxicol 2008 21 1 28 44 10.1021/tx700198a 17970581 PMC2602826

[B12] Doll R Morgan LG Speizer FE Cancers of the lung and nasal sinuses in nickel workers Br J Cancer 1970 24 4 623 632 5503591 10.1038/bjc.1970.76 PMC2008725

[B13] Kerckaert GA LeBoeuf RA Isfort RJ Use of the Syrian hamster embryo cell transformation assay for determining the carcinogenic potential of heavy metal compounds Fundam Appl Toxicol 1996 34 1 67 72 10.1006/faat.1996.0176 8937893

[B14] Kuper CF Woutersen RA Slootweg PJ Feron VJ Carcinogenic response of the nasal cavity to inhaled chemical mixtures Mutat Res 1997 380 1-2 19 26 9385386 10.1016/s0027-5107(97)00123-1

[B15] Miller AC Mog S McKinney L Luo L Allen J Xu J Page N Neoplastic transformation of human osteoblast cells to the tumorigenic phenotype by heavy metal-tungsten alloy particles: induction of genotoxic effects Carcinogenesis 2001 22 1 115 125 10.1093/carcin/22.1.115 11159749

[B16] Davidson TL Chen H Di Toro DM D'Angelo G Costa M Soluble nickel inhibits HIF-prolyl-hydroxylases creating persistent hypoxic signaling in A549 cells Mol Carcinog 2006 45 7 479 489 10.1002/mc.20176 16649251

[B17] Broday L Peng W Kuo MH Salnikow K Zoroddu M Costa M Nickel compounds are novel inhibitors of histone H4 acetylation Cancer Res 2000 60 2 238 241 10667566

[B18] Chen H Ke Q Kluz T Yan Y Costa M Nickel ions increase histone H3 lysine 9 dimethylation and induce transgene silencing Mol Cell Biol 2006 26 10 3728 3737 10.1128/MCB.26.10.3728-3737.2006 16648469 PMC1488989

[B19] Golebiowski F Kasprzak KS Inhibition of core histones acetylation by carcinogenic nickel(II) Mol Cell Biochem 2005 279 1-2 133 139 10.1007/s11010-005-8285-1 16283522

[B20] Kowara R Karaczyn A Cheng RY Salnikow K Kasprzak KS Microarray analysis of altered gene expression in murine fibroblasts transformed by nickel(II) to nickel(II)-resistant malignant phenotype Toxicol Appl Pharmacol 2005 205 1 1 10 10.1016/j.taap.2004.10.006 15885260

[B21] Karaczyn AA Golebiowski F Kasprzak KS Ni(II) affects ubiquitination of core histones H2B and H2A Exp Cell Res 2006 312 17 3252 3259 10.1016/j.yexcr.2006.06.025 16870173

[B22] Ke Q Davidson T Chen H Kluz T Costa M Alterations of histone modifications and transgene silencing by nickel chloride Carcinogenesis 2006 27 7 1481 1488 10.1093/carcin/bgl004 16522665

[B23] Klein CB Conway K Wang XW Bhamra RK Lin XH Cohen MD Annab L Barrett JC Costa M Senescence of nickel-transformed cells by an X chromosome: possible epigenetic control Science 1991 251 4995 796 799 10.1126/science.1990442 1990442

[B24] Klein CB Costa M DNA methylation, heterochromatin and epigenetic carcinogens Mutat Res 1997 386 2 163 180 10.1016/S1383-5742(96)00052-X 9113117

[B25] Lee YW Klein CB Kargacin B Salnikow K Kitahara J Dowjat K Zhitkovich A Christie NT Costa M Carcinogenic nickel silences gene expression by chromatin condensation and DNA methylation: a new model for epigenetic carcinogens Mol Cell Biol 1995 15 5 2547 2557 7537850 10.1128/mcb.15.5.2547 PMC230485

[B26] Ellen TP Kluz T Harder ME Xiong J Costa M Heterochromatinization as a potential mechanism of nickel-induced carcinogenesis Biochemistry 2009 48 21 4626 4632 10.1021/bi900246h 19338343 PMC2713359

[B27] Chen H Davidson T Singleton S Garrick MD Costa M Nickel decreases cellular iron level and converts cytosolic aconitase to iron-regulatory protein 1 in A549 cells Toxicol Appl Pharmacol 2005 206 3 275 287 10.1016/j.taap.2004.11.011 16039939

[B28] Costa M Davidson TL Chen H Ke Q Zhang P Yan Y Huang C Kluz T Nickel carcinogenesis: epigenetics and hypoxia signaling Mutat Res 2005 592 1-2 79 88 16009382 10.1016/j.mrfmmm.2005.06.008

[B29] Lu H Shi X Costa M Huang C Carcinogenic effect of nickel compounds Mol Cell Biochem 2005 279 1-2 45 67 10.1007/s11010-005-8215-2 16283514

[B30] Jin YH Dunlap PE McBride SJ Al-Refai H Bushel PR Freedman JH Global transcriptome and deletome profiles of yeast exposed to transition metals PLoS Genet 2008 4 4 e1000053 10.1371/journal.pgen.1000053 18437200 PMC2278374

[B31] Nishimura K Igarashi K Kakinuma Y Proton gradient-driven nickel uptake by vacuolar membrane vesicles of Saccharomyces cerevisiae J Bacteriol 1998 180 7 1962 1964 9537401 10.1128/jb.180.7.1962-1964.1998 PMC107116

[B32] Ruotolo R Marchini G Ottonello S Membrane transporters and protein traffic networks differentially affecting metal tolerance: a genomic phenotyping study in yeast Genome Biol 2008 9 4 R67 10.1186/gb-2008-9-4-r67 18394190 PMC2643938

[B33] Joho M Ikegami M Inohue H Tohoyama T Murayama T Nickel sensitivity of vacuolar membrane ATPase in a nickel resistant strain of Saccharomyces cerevisiae Biomed Lett 1993 48 115 120

[B34] Joho MIY Kunikane M Inohue H Tohoyama T The subcellular distribution of nickel ion in nickel-sensitive and ni-resistant strains of Saccharomyces cerevisiae Microbios 1992 71 149 159 1360616

[B35] Liu S Milne GT Kuremsky JG Fink GR Leppla SH Identification of the proteins required for biosynthesis of diphthamide, the target of bacterial ADP-ribosylating toxins on translation elongation factor 2 Mol Cell Biol 2004 24 21 9487 9497 10.1128/MCB.24.21.9487-9497.2004 15485916 PMC522255

[B36] Jorgensen R Merrill AR Yates SP Marquez VE Schwan AL Boesen T Andersen GR Exotoxin A-eEF2 complex structure indicates ADP ribosylation by ribosome mimicry Nature 2005 436 7053 979 984 10.1038/nature03871 16107839

[B37] Sahi C Craig EA Network of general and specialty J protein chaperones of the yeast cytosol Proc Natl Acad Sci USA 2007 104 17 7163 7168 10.1073/pnas.0702357104 17438278 PMC1855418

[B38] Honore B Rasmussen HH Celis A Leffers H Madsen P Celis JE The molecular chaperones HSP28, GRP78, endoplasmin, and calnexin exhibit strikingly different levels in quiescent keratinocytes as compared to their proliferating normal and transformed counterparts: cDNA cloning and expression of calnexin Electrophoresis 1994 15 3-4 482 490 10.1002/elps.1150150166 8055875

[B39] Verma R Ramnath J Clemens F Kaspin LC Landolph JR Molecular biology of nickel carcinogenesis: identification of differentially expressed genes in morphologically transformed C3H10T1/2 Cl 8 mouse embryo fibroblast cell lines induced by specific insoluble nickel compounds Mol Cell Biochem 2004 255 1-2 203 216 10.1023/B:MCBI.0000007276.94488.3d 14971661

[B40] Kuchin S Vyas VK Carlson M Snf1 protein kinase and the repressors Nrg1 and Nrg2 regulate FLO11, haploid invasive growth, and diploid pseudohyphal differentiation Mol Cell Biol 2002 22 12 3994 4000 10.1128/MCB.22.12.3994-4000.2002 12024013 PMC133850

[B41] Lamb TM Mitchell AP The transcription factor Rim101p governs ion tolerance and cell differentiation by direct repression of the regulatory genes NRG1 and SMP1 in Saccharomyces cerevisiae Mol Cell Biol 2003 23 2 677 686 10.1128/MCB.23.2.677-686.2003 12509465 PMC151549

[B42] Park SH Koh SS Chun JH Hwang HJ Kang HS Nrg1 is a transcriptional repressor for glucose repression of STA1 gene expression in Saccharomyces cerevisiae Mol Cell Biol 1999 19 3 2044 2050 10022891 10.1128/mcb.19.3.2044 PMC83997

[B43] Zhou H Winston F NRG1 is required for glucose repression of the SUC2 and GAL genes of Saccharomyces cerevisiae BMC Genet 2001 2 5 10.1186/1471-2156-2-5 11281938 PMC31344

[B44] Mason PB Struhl K Distinction and relationship between elongation rate and processivity of RNA polymerase II in vivo Mol Cell 2005 17 6 831 840 10.1016/j.molcel.2005.02.017 15780939

[B45] Rondon AG Jimeno S Garcia-Rubio M Aguilera A Molecular evidence that the eukaryotic THO/TREX complex is required for efficient transcription elongation J Biol Chem 2003 278 40 39037 39043 10.1074/jbc.M305718200 12871933

[B46] Chavez STB Rondon AG Erdjument-Bromage H Tempst P Svejstrup JQ Lithgow T Aguilera A A protein complex containing Tho2, Hpr1, Mft1 and a novel protein, Thp2, connects transcription elongation with mitotic recombination in Saccharomyces cerevisiae EMBO J 2000 19 5824 5834 10.1093/emboj/19.21.5824 11060033 PMC305808

[B47] Strasser K Masuda S Mason P Pfannstiel J Oppizzi M Rodriguez-Navarro S Rondon AG Aguilera A Struhl K Reed R TREX is a conserved complex coupling transcription with messenger RNA export Nature 2002 417 6886 304 308 10.1038/nature746 11979277

[B48] Zenklusen D Vinciguerra P Wyss JC Stutz F Stable mRNP formation and export require cotranscriptional recruitment of the mRNA export factors Yra1p and Sub2p by Hpr1p Mol Cell Biol 2002 22 23 8241 8253 10.1128/MCB.22.23.8241-8253.2002 12417727 PMC134069

[B49] Katzmann DJ Babst M Emr SD Ubiquitin-dependent sorting into the multivesicular body pathway requires the function of a conserved endosomal protein sorting complex, ESCRT-I Cell 2001 106 2 145 155 10.1016/S0092-8674(01)00434-2 11511343

[B50] Babst M Katzmann DJ Snyder WB Wendland B Emr SD Endosome-associated complex, ESCRT-II, recruits transport machinery for protein sorting at the multivesicular body Dev Cell 2002 3 2 283 289 10.1016/S1534-5807(02)00219-8 12194858

[B51] Yorikawa C Shibata H Waguri S Hatta K Horii M Katoh K Kobayashi T Uchiyama Y Maki M Human CHMP6, a myristoylated ESCRT-III protein, interacts directly with an ESCRT-II component EAP20 and regulates endosomal cargo sorting Biochem J 2005 387 Pt 1 17 26 15511219 10.1042/BJ20041227 PMC1134928

[B52] Kim J Sitaraman S Hierro A Beach BM Odorizzi G Hurley JH Structural basis for endosomal targeting by the Bro1 domain Dev Cell 2005 8 6 937 947 10.1016/j.devcel.2005.04.001 15935782 PMC2862258

[B53] Luhtala N Odorizzi G Bro1 coordinates deubiquitination in the multivesicular body pathway by recruiting Doa4 to endosomes J Cell Biol 2004 166 5 717 729 10.1083/jcb.200403139 15326198 PMC2172414

[B54] Scott A Chung HY Gonciarz-Swiatek M Hill GC Whitby FJ Gasper J Holton HM Viswanathan R Ghaffarian S Hill CP Structural and mechanistic studies of VPS4 proteins Embo J 2005 24 20 3658 3669 10.1080/00365590500240253 16193069 PMC1276703

[B55] Eide DJ Clark S Nair TM Gehl M Gribskov M Guerinot ML Harper JF Characterization of the yeast ionome: a genome-wide analysis of nutrient mineral and trace element homeostasis in Saccharomyces cerevisiae Genome Biol 2005 6 9 R77 10.1186/gb-2005-6-9-r77 16168084 PMC1242212

[B56] Shilatifard A Identification and purification of the Holo-ELL complex. Evidence for the presence of ELL-associated proteins that suppress the transcriptional inhibitory activity of ELL J Biol Chem 1998 273 18 11212 11217 10.1074/jbc.273.18.11212 9556611

[B57] Slagsvold T Pattni K Malerod L Stenmark H Endosomal and non-endosomal functions of ESCRT proteins Trends Cell Biol 2006 16 6 317 326 10.1016/j.tcb.2006.04.004 16716591

[B58] Pike BL Yongkiettrakul S Tsai MD Heierhorst J Mdt1, a novel Rad53 FHA1 domain-interacting protein, modulates DNA damage tolerance and G(2)/M cell cycle progression in Saccharomyces cerevisiae Mol Cell Biol 2004 24 7 2779 2788 10.1128/MCB.24.7.2779-2788.2004 15024067 PMC371128

[B59] Colgan DF Manley JL Mechanism and regulation of mRNA polyadenylation Genes Dev 1997 11 21 2755 2766 10.1101/gad.11.21.2755 9353246

[B60] Matsuoka S Ballif BA Smogorzewska A McDonald ER Hurov KE Luo J Bakalarski CE Zhao Z Solimini N Lerenthal Y ATM and ATR substrate analysis reveals extensive protein networks responsive to DNA damage Science 2007 316 5828 1160 1166 10.1126/science.1140321 17525332

[B61] Kemp HA Sprague GF Jr Far3 and five interacting proteins prevent premature recovery from pheromone arrest in the budding yeast Saccharomyces cerevisiae Mol Cell Biol 2003 23 5 1750 1763 10.1128/MCB.23.5.1750-1763.2003 12588993 PMC151714

[B62] Cordes VC Reidenbach S Rackwitz HR Franke WW Identification of protein p270/Tpr as a constitutive component of the nuclear pore complex-attached intranuclear filaments J Cell Biol 1997 136 3 515 529 10.1083/jcb.136.3.515 9024684 PMC2134304

[B63] Liao H Winkfein RJ Mack G Rattner JB Yen TJ CENP-F is a protein of the nuclear matrix that assembles onto kinetochores at late G2 and is rapidly degraded after mitosis J Cell Biol 1995 130 3 507 518 10.1083/jcb.130.3.507 7542657 PMC2120529

[B64] Hartwig A Asmuss M Ehleben I Herzer U Kostelac D Pelzer A Schwerdtle T Burkle A Interference by toxic metal ions with DNA repair processes and cell cycle control: molecular mechanisms Environ Health Perspect 2002 110 Suppl 5 797 799 12426134 10.1289/ehp.02110s5797 PMC1241248

[B65] Ke Q Li Q Ellen TP Sun H Costa M Nickel compounds induce phosphorylation of histone H3 at serine 10 by activating JNK-MAPK pathway Carcinogenesis 2008 29 6 1276 1281 10.1093/carcin/bgn084 18375956 PMC2829883

[B66] Ouyang W Zhang D Li J Verma UN Costa M Huang C Soluble and insoluble nickel compounds exert a differential inhibitory effect on cell growth through IKKalpha-dependent cyclin D1 down-regulation J Cell Physiol 2009 218 1 205 214 10.1002/jcp.21590 18792914 PMC2605425

[B67] Haugen AC Kelley R Collins JB Tucker CJ Deng C Afshari CA Brown JM Ideker T Van Houten B Integrating phenotypic and expression profiles to map arsenic-response networks Genome Biol 2004 5 12 R95 10.1186/gb-2004-5-12-r95 15575969 PMC545798

[B68] Serero A Lopes J Nicolas A Boiteux S Yeast genes involved in cadmium tolerance: Identification of DNA replication as a target of cadmium toxicity DNA Repair (Amst) 2008 7 8 1262 1275 10.1016/j.dnarep.2008.04.005 18514590

[B69] Thorsen M Perrone GG Kristiansson E Traini M Ye T Dawes IW Nerman O Tamas MJ Genetic basis of arsenite and cadmium tolerance in Saccharomyces cerevisiae BMC Genomics 2009 10 105 10.1186/1471-2164-10-105 19284616 PMC2660369

[B70] Altschul SF Gish W Miller W Myers EW Lipman DJ Basic local alignment search tool J Mol Biol 1990 215 3 403 410 2231712 10.1016/S0022-2836(05)80360-2

[B71] Robinson MD Grigull J Mohammad N Hughes TR FunSpec: a web-based cluster interpreter for yeast BMC Bioinformatics 2002 3 35 10.1186/1471-2105-3-35 12431279 PMC139976

[B72] Lee TI Rinaldi NJ Robert F Odom DT Bar-Joseph Z Gerber GK Hannett NM Harbison CT Thompson CM Simon I Transcriptional regulatory networks in Saccharomyces cerevisiae Science 2002 298 5594 799 804 10.1126/science.1075090 12399584

[B73] Rooney JP George AD Patil A Begley U Bessette E Zappala MR Huang X Conklin DS Cunningham RP Begley TJ Systems based mapping demonstrates that recovery from alkylation damage requires DNA repair, RNA processing, and translation associated networks Genomics 2009 93 1 42 51 10.1016/j.ygeno.2008.09.001 18824089 PMC2633870

[B74] Said MR Begley TJ Oppenheim AV Lauffenburger DA Samson LD Global network analysis of phenotypic effects: protein networks and toxicity modulation in Saccharomyces cerevisiae Proc Natl Acad Sci USA 2004 101 52 18006 18011 10.1073/pnas.0405996101 15608068 PMC539745

[B75] Shannon P Markiel A Ozier O Baliga NS Wang JT Ramage D Amin N Schwikowski B Ideker T Cytoscape: a software environment for integrated models of biomolecular interaction networks Genome Res 2003 13 11 2498 2504 10.1101/gr.1239303 14597658 PMC403769

