# Supplementary material for: A genome-wide deletion mutant screen identifies pathways affected by nickel sulfate in Saccharomyces cerevisiae
Source: BMC Genomics. 2009 Nov 15;10:524. doi: 10.1186/1471-2164-10-524 (PMC2784802; doi:10.1186/1471-2164-10-524)
Supplement: Additional file 3 — Functional categories overrepresented with proteins (that have a similar human protein) whose absence renders cells more sensitive to NiSO4. Functional categories overrepresented with proteins whose absence renders cells sensitive to nickel were identified using FunSpec. [file 1471-2164-10-524-S3.PPT]

## Slide 1
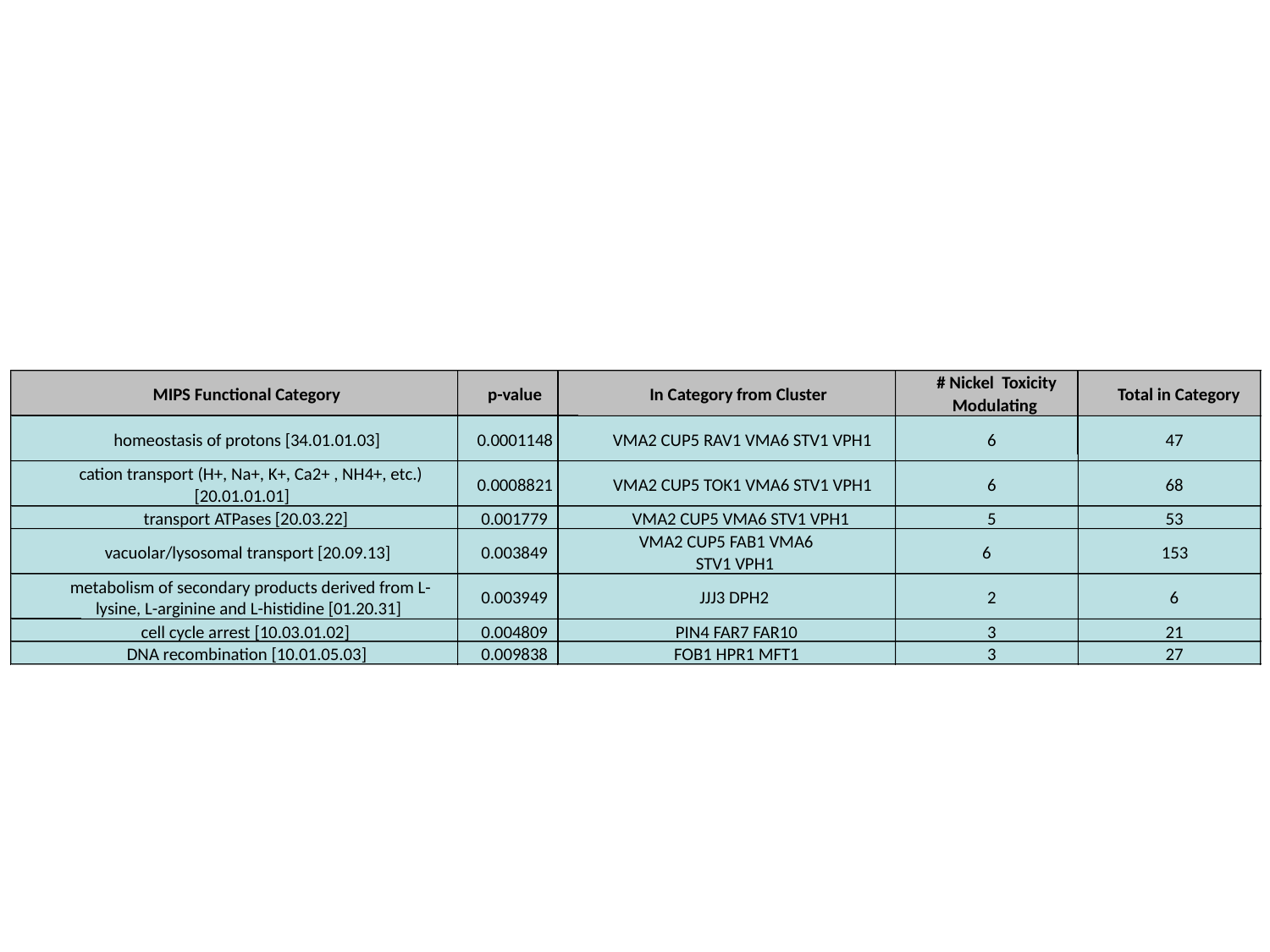

# Nickel Toxicity
MIPS Functional Category
p-value
In Category from Cluster
Total in Category
Modulating
homeostasis of protons [34.01.01.03]
0.0001148
VMA2 CUP5 RAV1 VMA6 STV1 VPH1
6
47
cation transport (H+, Na+, K+, Ca2+ , NH4+, etc.)
0.0008821
VMA2 CUP5 TOK1 VMA6 STV1 VPH1
6
68
[20.01.01.01]
transport ATPases [20.03.22]
0.001779
VMA2 CUP5 VMA6 STV1 VPH1
5
53
VMA2 CUP5 FAB1 VMA6
vacuolar/lysosomal transport [20.09.13]
0.003849
6
153
STV1 VPH1
metabolism of secondary products derived from L-
0.003949
JJJ3 DPH2
2
6
lysine, L-arginine and L-histidine [01.20.31]
cell cycle arrest [10.03.01.02]
0.004809
PIN4 FAR7 FAR10
3
21
DNA recombination [10.01.05.03]
0.009838
FOB1 HPR1 MFT1
3
27
