# Supplementary figures and images for: A genome-wide deletion mutant screen identifies pathways affected by nickel sulfate in Saccharomyces cerevisiae
Source: BMC Genomics. 2009 Nov 15;10:524. doi: 10.1186/1471-2164-10-524 (PMC2784802; doi:10.1186/1471-2164-10-524)

## Slide 1
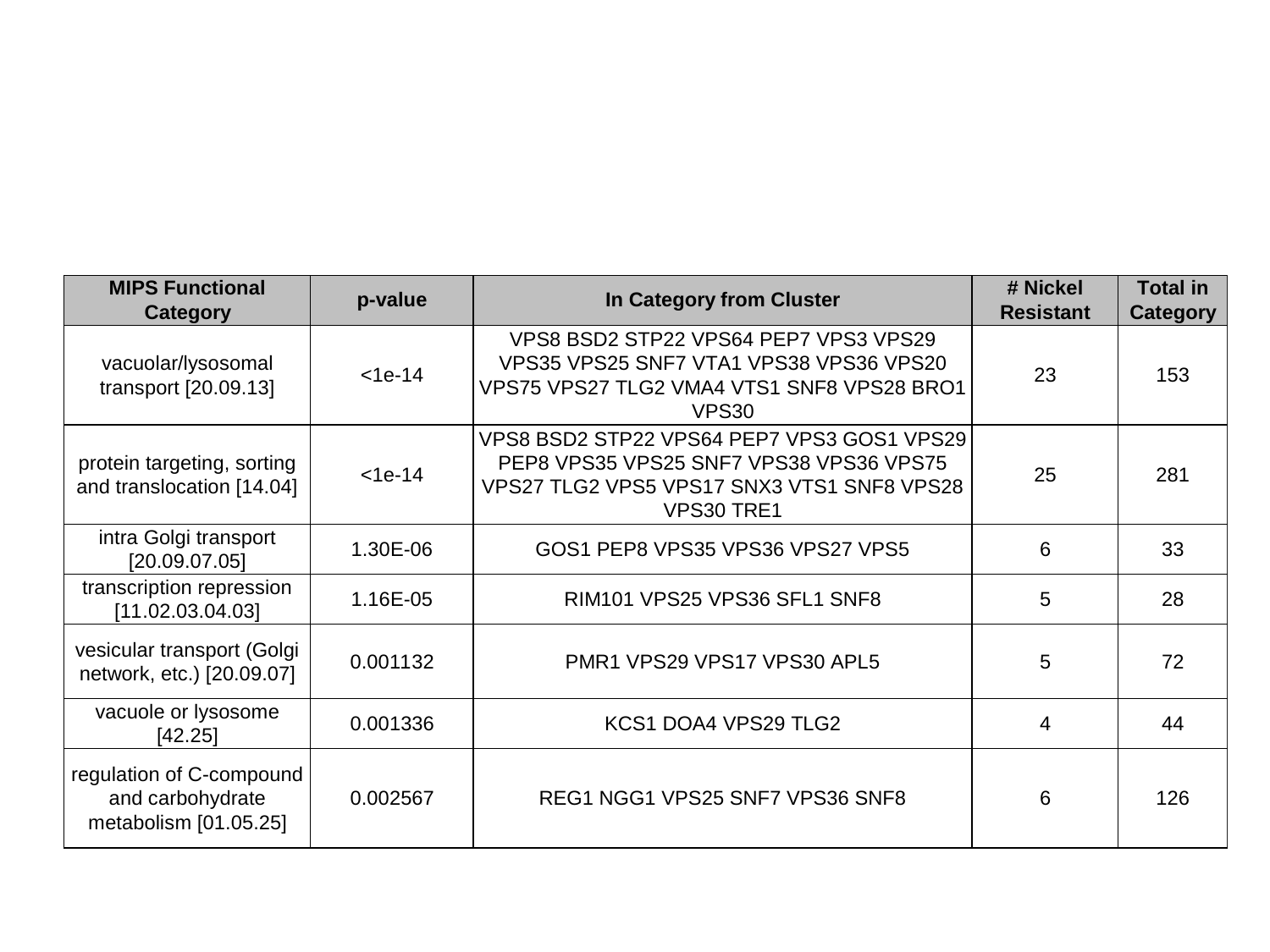

Supplement: Additional file 4 — Functional categories overrepresented with proteins (that have a similar human protein) whose absence renders cells more resistant to NiSO4. Functional categories overrepresented with proteins whose absence renders cells resistant to nickel were identified using FunSpec. [file 1471-2164-10-524-S4.PPT]

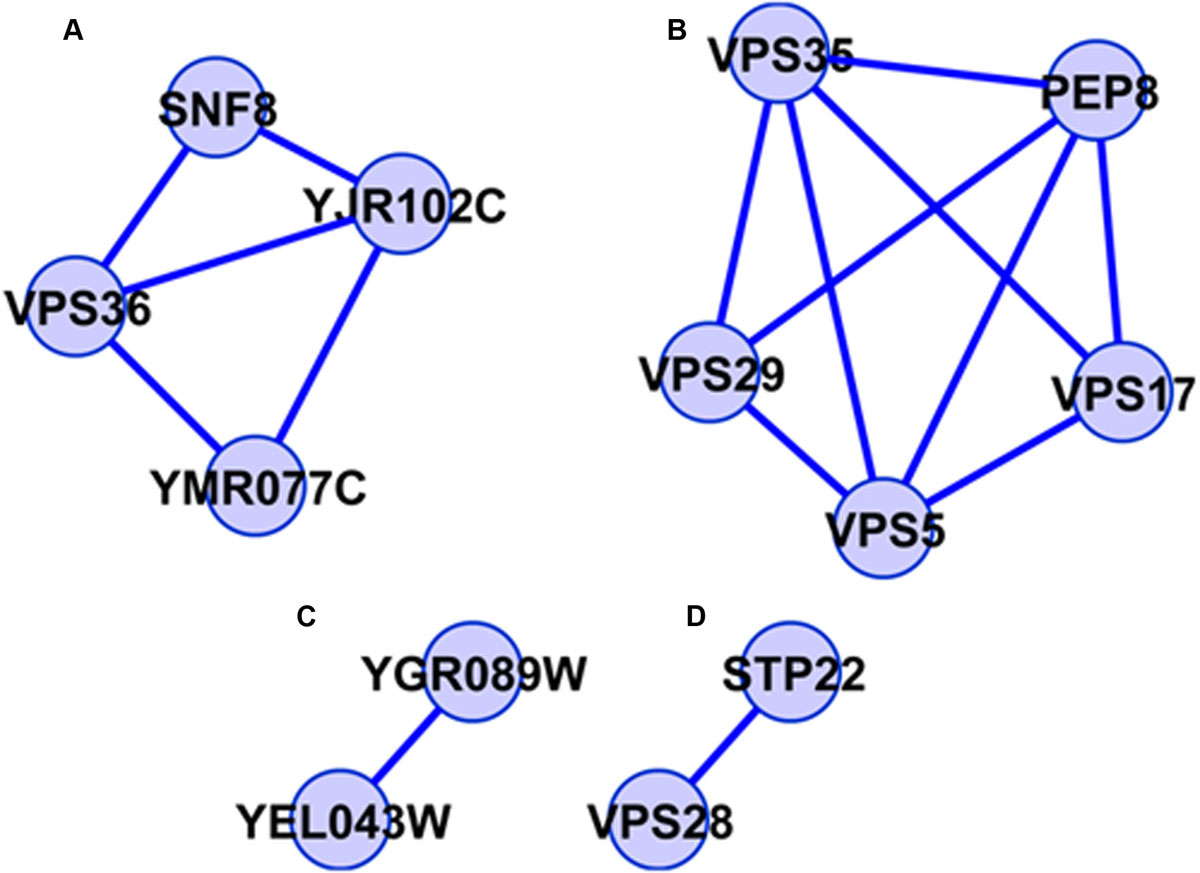

Supplement: Additional file 5 — Nickel toxicity modulating networks identified with proteins (that have a similar human protein) whose absence renders cells more sensitive to NiSO4. The yeast protein interactome consisting of 5,433 proteins, 14,656 protein-protein interactions, and 5,621 protein-DNA interactions was compiled using the program Cytoscape. Proteins corresponding to nickel sensitive gene deletion strains were mapped onto the interactome and then filtered to identify connected groups of proteins (N => 2). Straight lines indicate protein-protein interactions. [file 1471-2164-10-524-S5.JPEG]

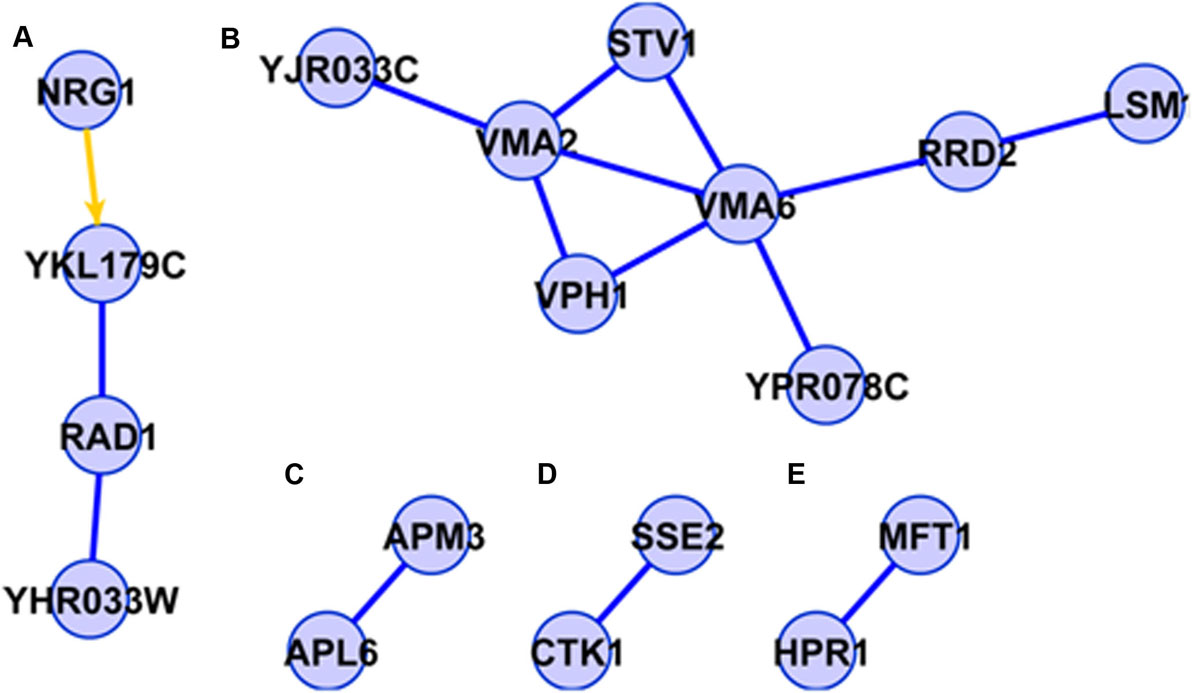

Supplement: Additional file 6 — Nickel resistance networks identified with proteins (that have a similar human protein) whose absence renders cells more resistant to NiSO4. The yeast protein interactome consisting of 5,433 proteins, 14,656 protein-protein interactions, and 5,621 protein-DNA interactions was compiled using the program Cytoscape. Proteins corresponding to nickel resistant gene deletion strains were mapped onto the interactome and then filtered to identify connected groups of proteins (N => 2). Straight lines indicate protein-protein interactions and arrows indicate DNA-protein interaction. [file 1471-2164-10-524-S6.JPEG]
